# Role of Uncoupling Protein 2 Gene Polymorphisms on the Risk of Ischemic Stroke in a Sardinian Population

**DOI:** 10.3390/life12050721

**Published:** 2022-05-12

**Authors:** Rosita Stanzione, Maria Cotugno, Maurizio Forte, Franca Bianchi, Simona Marchitti, Nicole Piera Palomba, Teresa Esposito, Bastianina Zanda, Alessandra Sanna, Speranza Rubattu

**Affiliations:** 1 IRCCS Neuromed, Località Camerelle, 86077 Pozzilli, Italy; maria.cotugno@neuromed.it (M.C.); maurizio.forte@neuromed.it (M.F.); franca.bianchi@neuromed.it (F.B.); simona.marchitti@neuromed.it (S.M.); nicole.palomba.89@gmail.com (N.P.P.); teresa.esposito@igb.cnr.it (T.E.); 2 Institute of Genetic and Biophysics “Adriano Buzzati Traverso”, Italian National Research Council (CNR), 80131 Naples, Italy; 3 Stroke Unit, SS. Annunziata Hospital, 07100 Sassari, Italy; bastianina.zanda@gmail.com (B.Z.); alessandra.sanna@aousassari.it (A.S.); 4 Clinical and Molecular Medicine Department, School of Medicine and Psychology, Sapienza University of Rome, 00189 Rome, Italy

**Keywords:** vascular disease, ischemic stroke, uncoupling protein 2, genetic association, Sardinians

## Abstract

The mitochondrial uncoupling protein 2 (UCP2) acts as an anion transporter and as an antioxidant factor able to reduce the reactive oxygen species level. Based on its effects, UCP2 prevents the membrane lipids, proteins, and DNA damage while preserving normal cellular functions. Many variants have been identified within the human *UCP2*. Some of them were associated with a higher risk of obesity, diabetes and cardiovascular diseases in different populations. *UCP2* appears a suitable candidate also for the risk of ischemic stroke. In the current study, we investigated the possible association between few variants of *UCP2* (rs659366, rs660339, rs1554995310) and the risk of ischemic stroke in a genetically homogenous cohort of cases and controls selected in Sardinia Island. This population has been previously analysed for other candidate genes. A total of 250 cases of ischemic stroke and 241 controls were enrolled in the study. The allelic/genotypic distribution of the 3 *UCP2* variants was characterized and compared among cases and controls. The results of our study confirmed known risk factors for ischemic stroke: age, history of smoking, hypertension, hypercholesterolemia, and atrial fibrillation. No association was found between the 3 *UCP2* variants and the risk of ischemic stroke in our Sardinian cohort.

## 1. Introduction

Ischemic stroke (IS) is a multifactorial disease caused by the contribution of both modifiable (environmental and behavioral) and non-modifiable factors, the latter including genetic factors [1,2,3]. Many studies conducted in recent years have shown, both in animal models and in human populations, the contribution of specific genes to the occurrence of IS. However, a complete knowledge of all genes involved in the occurrence of human stroke is a difficult task to achieve.

The gene encoding uncoupling protein 2 (UCP2) represents a suitable candidate for the etiopathogenesis of many vascular diseases, including stroke [4,5,6]. UCP2, belonging to the family of uncoupling proteins (UCPs), is encoded by a gene mapping on human chromosome 11 (11q13.4). Among the five identified uncoupling proteins, UCP2 is widely expressed in nearly all mammalian tissues, including white adipose tissue, liver, kidney, pancreatic islets, macrophages, and retinal endothelial cells. At the cellular level, the UCP2 protein is located within the inner mitochondrial membrane, where it acts as an anion transporter and as an antioxidant factor able to reduce the reactive oxygen species (ROS) accumulation [7,8]. Due to its functions, UCP2 maintains the integrity of the mitochondria and avoids the damage of the membrane lipids, proteins, and DNA, therefore preserving cellular health [8]. A large amount of evidence, obtained both in animal models and in humans, revealed that UCP2 is involved in the pathogenesis of metabolic, cardiovascular, and neurodegenerative diseases [8]. UCP2 expression is increased in type 2 diabetes mellitus (T2DM), where it inhibits insulin secretion from pancreatic β-cells [8,9,10]. High UCP2 levels exert a protective effect in heart diseases [11,12], atherosclerosis [13,14], and IS [4,15,16], whereas low UCP2 levels exert a detrimental effect [16]. Previous studies from our group highlighted a key role of UCP2 in the predisposition to stroke of the stroke-prone spontaneously hypertensive rat (SHRSP), a suitable model of human disease. Specifically, we showed that reduced UCP2 expression associated with cerebral and renal vascular damage in the high-salt-fed SHRSP and that UCP2 overexpression reduced inflammation and oxidative stress leading to a significant protection from the vascular injury occurrence in the same model [5,6,17,18].

Several single-nucleotide polymorphisms (SNPs) have been identified within the human *UCP2* [19]. The rs659366 SNP (−866G/A), located in the promoter region, is involved in the modulation of gene expression [20,21]. The rs660339 SNP (Ala55Val), located within exon 4, appears able to interfere with UCP2 phosphorylation by protein kinase C (PKC), reducing its activity [22,23]. The third variant, rs1554995310, is a 45 bp insertion/deletion (ins/del) located at the 3′ end of exon 8 of the gene. The homozygosity for the insertion was associated with reduced gene transcription and reduced mRNA stability [22,24]. These variants were associated with increased occurrence of obesity and diabetes [22,25,26], with an increased risk of IS in Chinese women affected by T2DM [27,28], and with embolic IS after recanalization [29].

In the present study, we investigated the potential association between the three *UCP2* variants and the risk of IS in a genetically homogenous population from the northern Sardinia Island, Italy.

## 2. Results

The clinical characteristics of the study population are shown in the Table 1. Older age, hypercholesterolemia, hypertension, and atrial fibrillation were significantly associated with the presence of IS as obtained by the Fisher’s exact test.

**Figure 1:**
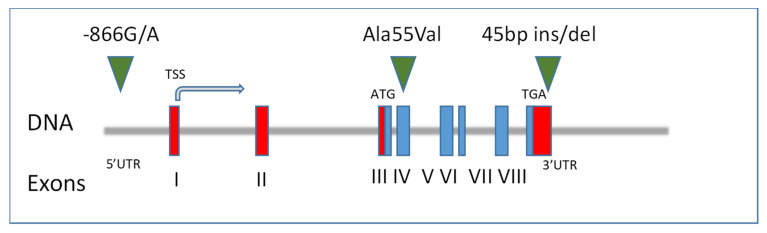
Genomic organization of *UCP2* (Chr11q13) and the localization of the three variants considered in the study. Human *UCP2* is localized on chromosome 11 (11q13). The transcription start site (TSS) of the gene is indicated by an arrow. The non-translated (red boxes) and the translated (blue boxes) exons are shown on the scheme. ATG and TGA are the start and stop codons, respectively. The positions of three *UCP2* variants (−866G/A, Ala55Val, 45 bp ins/del) are also indicated in the figure.

In this cohort, we studied three variants distributed along *UCP2*: rs659366 (−866G/A, 5′UTR), rs660339 (Ala55Val, exon 4), and rs1554995310, an insertion/deletion of 45 bp at the 3′ end of exon 8 (Figure 1).

A strong linkage disequilibrium (LD) was detected between the rs660339 and rs659366 SNPs (r^2^ 0.84, D’0.99). Therefore, the association analysis was performed by using data from the rs659366 and rs1554995310 SNPs.

Genotype and allele frequencies for the two *UCP2* variants in cases and controls are summarized in Table 2. The variants were in Hardy–Weinberg equilibrium (HWE). The minor allele frequencies for both variants were like those described in other Caucasian populations.

We did not observe any significant difference in the distribution of alleles and genotypes of the two variants between cases and controls.

The Table 3 shows the independent risk factors for IS in the analyzed population as obtained by a logistic regression analysis. The analysis showed a significant correlation between known risk factors, such as age, history of smoking, hypercholesterolemia, hypertension, and atrial fibrillation, with the risk of IS. In contrast, no correlation between genotypes, risk factors, and occurrence of IS was found in this cohort.

## 3. Discussion

In the present study, we examined the association between known *UCP2* variants and the risk of IS in a genetically homogeneous population from the Sardinia Island. The *UCP2* variants were selected based on previous evidence reporting significant associations with IS [28,29,30] or with other cardiovascular diseases [31,32,33,34].

Typical risk factors for IS were confirmed in this cohort. In particular, older age, history of smoking, hypertension, hypercholesterolemia, atrial fibrillation were significantly associated with increased IS occurrence. However, no evidence of association between the *UCP2* variants and the risk of IS was found in this Sardinian population.

The UCP2 protein is encoded by a gene mapping on human chromosome 11, in a region linked to energy homeostasis and obesity [35]. Several variants located in the promoter, exon, and intronic regions of the gene are known. In particular, the −866G/A single nucleotide polymorphism, located within the promoter region of the gene, is a functional variant. The common G allele was associated by both in vivo and in vitro evidence with a reduction of UCP2 mRNA expression, increased ROS production [36], increased risk of obesity, and, at the same time, reduced risk of T2DM [24,37,38]. Conversely, the AA genotype was associated with increased UCP2 expression [24,39]. Of note, Díaz-Maroto Cicuéndez et al. highlighted the role of the −866G/A polymorphism as a marker of functional prognosis of IS after recanalization. In particular, the authors suggested a “good predictor” role of the AA genotype compared to AG or GG genotypes in this condition [29]. Moreover, the G allele variant correlated with an increased risk of IS in Chinese women affected by T2DM in a 4-year prospective study [27,28].

The common Ala55Val polymorphism (rs660339 G/A) has been variably associated with occurrence of obesity [19], insulin resistance [26], T2DM [40], and cardiovascular diseases [31] in many populations. However, other studies were unable to detect a role of Ala55Val in atherosclerosis [41] or metabolic syndrome [42].

The 45 bp insertion/deletion was previously associated with inflammation [43], metabolic syndrome [26,44], obesity [45], renal failure [46], and T2DM [33]. Only one study reported a significant correlation between the 45 bp ins/del of *UCP2* with hyperhomocysteinemia, a known risk factor for IS, in a Caucasian population. Hyperhomocysteinemia, in turn, might increase the risk of venous thrombosis and promote the development of cerebral ischemic lesions [47].

Mattiasson et al. demonstrated that UCP2 overexpression might exert a neuroprotective effect both in vivo and in vitro conditions. In fact, UCP2 overexpression reduced ROS level and brain damage while preserving neurological functions in the transient middle-cerebral artery occlusion (MCAO) transgenic mice. In addition, UCP2 overexpression preserved the mitochondrial membrane potential, leading to a reduction of oxidative stress, cell death, and pro-apoptotic factors release in cultured cortical neurons exposed to glucose and oxygen deprivation [48]. Our research group demonstrated that the downregulation of UCP2 associated with increased occurrence of renal and cerebrovascular injury in SHRSP rats fed with a Japanese-style diet. On the other hand, UCP2 overexpression, upon administration of either Brassica oleracea (BO) sprout extract or fenofibrate, protected from the occurrence of cerebrovascular and renal damage this rat model [5,6].

Interestingly, our current study was performed in a sample from the northern Sardinia Island, which is regarded as a “genetic isolate”. In fact, Sardinians retain ancient genetic variants currently very rare in other Italian regions or continental Europe. Due to the peculiar distribution of the different genetic variants, and to the numerous genetic-based diseases (including immune type 1 diabetes) particularly frequent in the island, this population has always aroused considerable interest in human genetics [49,50]. In addition, our research group has previously investigated the contributory role of several candidate genes into the susceptibility to develop IS of this Sardinian cohort. In fact, we demonstrated a direct contributory role to IS occurrence for the following gene variants: atrial natriuretic peptide (C2238T) [51], type 1 angiotensin II receptor (A1166C) [52], Factor VII (-C122T) [53], cholesteryl ester transfer protein Taq1 B1/B2) [54], β2-adrenergic receptor (Gln27Glu) [55], and platelet glycoprotein IIIa (PlA1/A2) [56]. Conversely, we found no evidence of association for the phosphodiesterase-4D (PDE4D) and 5-lipoxygenase activating protein (ALOX5AP) gene variants with the risk of IS [57]. Overall, the previous results support a suitability of the selected population for the analysis of candidate genes in the pathogenesis of IS.

The results of the present study do not support the working hypothesis of a role of *UCP2* into the risk of IS in the Sardinian sample. A limitation of the current study may be represented by the smaller sample size of cases and controls used for the analysis. To explore further the role of *UCP2* as a potential contributory gene for the risk of IS in Sardinians, future investigations should test its role by using a larger population size.

## 4. Materials and Methods

### 4.1. Study Population

The subjects analyzed in this study included 250 cases and 241 controls. Cases were recruited at the Department of Neurology of the University of Sassari (Sassari, Sardinia, Italy) between September 1998 and March 2003. The diagnosis of ischemic stroke was based on clear, unequivocal clinical parameters, with signs and symptoms persisting for more than 24 h and confirmed by computed tomography scan or nuclear magnetic resonance imaging. Stroke subtypes were as follows: large-vessel disease (47%), small-artery disease (27%), embolic stroke (24%), other origins, and unknown (2%). The time from stroke occurrence to hospital recovery was within 48 h. At hospital admission, NIHSS classification ranged from minor (20%) to moderate (40%) and severe stroke (40%). The size of the infarcted brain area varied depending on the type of stroke and commonly involved about two-thirds of the hemisphere in the severe form of stroke (because of the middle cerebral artery occlusion). Another common localization in the embolic subtype of stroke was within the brainstem and cerebellum.

A venous whole blood sample was always performed for genomic DNA extraction few days after the cerebrovascular event. No plasma samples were collected. Notably, at the time of stroke patients’ recruitment, treatments with tissue plasminogen activator and thrombectomy were at their beginning and still not utilized in the Sardinian center. The therapies commonly administered to the patients were based on antiplatelet drugs (clopidogrel and acetylsalicylic acid) and low molecular weight heparin.

Unrelated control subjects from the same geographical area were randomly selected among patients admitted to the same hospital, mainly from surgical, urological, dermatological, and ophthalmological inpatient and outpatient clinics. They were included if they had vascular risk factors or a history of cardiovascular disease (myocardial infarction, previous coronary revascularization procedures, and peripheral vascular disease), but they were excluded if they had either current or previous cerebrovascular disease. Consanguineous subjects were excluded based on an accurate and careful family history of each individual.

Hypertension was defined as present if subjects had been previously diagnosed according to World Health Organization/International Society of Hypertension guidelines and were routinely receiving anti-hypertensive therapy. History of smoking was defined when subjects had stopped smoking 2 months before the study. Hypercholesterolemia was defined as a total cholesterol blood level >220 mg/dL. Alcohol consumption was defined as an intake of >30 g/day. The presence or absence of diabetes, history of myocardial infarction, and peripheral arteriopathy was recorded.

### 4.2. Ethical Approval

All procedures performed in this study were in accordance with the ethical standards of the institutional research committee at the Sassari University and with the 1964 Helsinki declaration and its later amendments or comparable ethical standards.

### 4.3. DNA Isolation and Genotyping

Genomic DNA was isolated from peripheral blood using a commercially available kit (QIAamp DNA Blood Mini kit, Qiagen, Hilden, Germany). 

All *UCP2* variants except the 45 bp ins/del were genotyped by the method of allelic discrimination in real-time polymorphism chain reaction using Predesigned TaqMan SNP Genotyping Assays and TaqMan™ Genotyping Master Mix (Applied Biosystems, Waltham, MA, USA). Genotyping reactions were conducted on ViiA 7 Real-Time PCR System (Thermo Fisher Scientific, Waltham, MA, USA). The 45 bp ins/del variant of the *UCP2* gene was detected by standard electrophoretic separation of PCR products. For this purpose, 100 ng of template DNA and 10 µM of forward (5′-CAGTGAGGGAAGTGGGAGGG-3′) and reverse (5′-GGGGCAGGACGAAGATTC-3′) primers were mixed, and the volume was made up to 12.5 µL with sterile water. The samples were subjected to a PCR reaction using Amplitaq DNA polymerase reagents (Thermo Fisher Scientific) and processed on a T100 Thermal Cycler (Bio-Rad, Hercules, CA, USA). PCR products were resolved on agarose gels and visualized by Syber safe agarose gel (Thermo Fisher Scientific) staining and ultraviolet illumination (ChemiDoc Imaging Systems, Bio-Rad). The presence of a 457 bp product indicated the *Ins* allele, whereas the presence of a 412 bp product indicated the *Del* allele. Each DNA sample revealed 1 of the 3 possible patterns after electrophoresis: a 412 bp band (genotype DD), a 457 bp band (genotype II), or both (genotype DI) [58].

### 4.4. Statistical Analysis

Age was expressed as median value with inter-quartile range (IQR). Categorical variables were compared with χ^2^ test or Fisher’s exact test as appropriate. Genotype and allele frequencies were computed and their distribution in cases and controls was analyzed by χ^2^ test with 2 DF and 1 DF. Concordance to the frequency predicted by the Hardy–Weinberg equilibrium (HWE) was assessed by χ^2^ test with 1 DF. The risk associated with each genotype to the occurrence of IS was estimated by logistic regression analysis under the assumption of an additive inheritance model (i.e., fitting the three genotypes assuming one-step increase in odds per mutant allele). A multivariable logistic model was carried out including the genetic predictor and potential confounders. In particular, the multivariate model included variables that were significant (*p* < 0.2) in the univariate analysis, any potential confounder that changed the unadjusted OR for genotype by >5% after adjustment or earlier-recognized risk factors associated with IS. Based on these criteria, age (added to the model as continuous variable), gender, history of smoking, diabetes, hypercholesterolemia, hypertension, and atrial fibrillation were included in the model. Likelihood ratio tests were used to assess the significance of the model.

SPSS statistical software (SPSS Inc., Chicago, IL, USA, version 12.0) was used for most of the statistical analysis. Tests for deviation from Hardy−Weinberg equilibrium and tests for association were performed with DeFinetti (http://ihg.gsf.de/cgi-bin/hw/hwa1.pl, accessed on 13 February 2022). The difference between the observed means was calculated with MedCalc (https://www.medcalc.org/calc/comparison_of_means.php, accessed on 13 February 2022). The Linkage Disequilibrium (LD) Calculator was used for calculating LD between variants (https://grch37.ensembl.org/Homo_sapiens/Tools/LD?db=core;expand_form=true;tl=aZGFAujRCzabrjTT-8016950, accessed on 13 February 2022). The power of our case-control sample was calculated by QUANTO statistical software. This study had the ability to detect, assuming 80% power and a two-sided 0.05 significance level with a minor allele frequency of 20%, an odds ratio > 1.51 (https://www.stat.ubc.ca/~rollin/stats/ssize/caco.html, accessed on 13 February 2022). All calculations were considered significant for a *p*-value < 0.05.

## Figures and Tables

**Table 1:** Baseline characteristics of ischemic stroke cases and controls.

Variables	Controls (N = 241)	Ischemic Strokes (N = 250)	Odds Ratio (95% CI)	*p*-Value
**Median age in years ^a^**	69.57 (SD13.9)	74.06 (SD11.39)	1.145 (2.24–6.73)	**0.0001**
**Sex, Males**	138 (57.2%)	149 (59.6%)	1.10 (0.76–1.57)	0.59
**History of smoking**	89 (36.9%)	98 (39.2%)	1.10 (0.76–1.8)	0.6
**Alcohol**	32 (13.2%)	30 (12.0%)	0.89 (0.52–1.51)	0.67
**Diabetes**	68 (28.2%)	59 (23.6%)	0.78 (0.52–1.17)	0.24
**Hypercholesterolemia ^a^**	29 (12.0%)	58 (23.2%)	2.20 (1.35–3.59)	**0.001**
**Peripheral arteriopathy**	13 (5.4%)	10 (4.0%)	0.73 (0.31–1.69)	0.46
**Hypertension ^a^**	114 (47.3%)	164 (65.6%)	2.28 (1.58–3.28)	**<0.0001**
**History of MI**	34 (14.1%)	38 (15.2%)	1.09 (0.66–1.80)	0.72
**Atrial fibrillation** **^a^**	37 (15.3%)	68 (27.2%)	2.06 (1.31–3.2)	**0.001**

Values are expressed as mean ± SD or N of subjects (with the corresponding percentages). ^**a**^ Indicates variables which are significantly different between cases and control groups. MI, myocardial infarction.

**Table 2:** Genotype and allele distribution of *UCP2* variants in cases and controls.

Markers	CNT (N = 241)	Stroke (N = 250)	Odds Ratio (CI 95%)	*p*-Value
**rs659366**				
(−866G/A)				
	GG 114 (47, 3%)	GG 118 (47, 2%)		
**Genotype N (%)**	GA 104 (43, 1%)	GA 110 (44%)		0.80
	AA 23 (9, 6%)	AA 22 (8, 8%)	0.92 (0.48–1.75)	
**Allele N (%)**	G 332 (68, 9%)	G 346 (69.2%)		
	A 150 (31.1%)	A 154 (30.8%)	0.98 (0.752–1.291)	0.91
**rs1554995310**				
(45 bp ins/del)				
	DD 134 (55, 6%)	DD 141 (56, 4%)		
**Genotype N (%)**	DI 91 (37, 8%)	DI 97 (38, 8%)		0.39
	II 16 (6, 6%)	II 12 (4, 8%)	0.71 (0.32–1.56)	
**Allele N (%)**	D 359 (74.5%)	D 379 (78.6%)		
	I 123 (25.5%)	I 121 (21.4%)	0.93 (0.698–1.245)	0.93

CNT, controls; CI, confidence interval; DD, homozygous for deletion; DI, heterozygous; II, homozygous for insertion.

**Table 3:** Independent risk factors for IS by logistic regression analysis.

Variables	Coefficient	SE	*p*-Value	OR	CI 95%
**rs659366** (−866G/A)	0.0124	0.0376	0.7415	1.0125	(0.9405–1.0900)
**rs1554995310** (45 bp ins/del)	−0.049	0.0466	0.2936	0.9522	(0.8691–1.0433)
**Sex**	0.0211	0.2235	0.9247	1.0213	(0.6591–1.5826)
Age *	0.0233	0.0089	**0.009**	**1.0236**	(1.0058–1.0416)
**History of smoking**	0.8277	0.2963	**0.0052**	**2.2879**	(1.2802–4.0891)
**Alcohol**	0.4991	0.3553	0.1601	**1.6473**	(0.8209–3.3054)
**Diabetes**	−0.3551	0.2267	0.1172	0.7011	(0.4496–1.0933)
**Hypercholesterolemia**	0.848	0.2708	**0.0017**	**2.3351**	(1.3735–3.9698)
**Peripheral arteriopathy**	−0.2036	0.4755	0.6685	0.8158	(0.3212–2.0715)
**Hypertension**	0.6093	0.2031	**0.0027**	**1.8391**	(1.2352–2.7382)
**History of MI**	−0.1287	0.2785	0.644	0.8792	(0.5093–1.5177)
**Atrial fibrillation**	0.5975	0.2468	**0.0155**	**1.8176**	(1.1206–2.9481)
**Constant**	−1.8374	0.9315	0.0485		

Model Fit: Chi-Square = 65.9360. df = 13; *p*-value = 0.0000. SE, standard error; OR, odds ratio; CI, confidence interval. * Considered as continuous variable.

## Data Availability

The data presented in this study are available on request from the corresponding author.
